# Asparagine Repeats in *Plasmodium falciparum* Proteins: Good for Nothing?

**DOI:** 10.1371/journal.ppat.1003488

**Published:** 2013-08-22

**Authors:** Vasant Muralidharan, Daniel E. Goldberg

**Affiliations:** 1 Center for Tropical & Emerging Global Diseases and Department of Cellular Biology, University of Georgia, Athens, Georgia, United States of America; 2 Howard Hughes Medical Institute, Departments of Medicine and Molecular Microbiology, Washington University School of Medicine, St. Louis, Missouri, United States of America; University of Wisconsin Medical School, United States of America

Malaria is a deadly parasitic human disease that poses a significant health risk for about 3.3 billion people in the tropical and subtropical regions of the world [1]. The past decade has seen significant progress in our understanding of the biology of the most deadly parasite species, *Plasmodium falciparum*. The groundwork for this progress was laid by genome sequencing efforts that revealed a number of surprising features [2], [3]. One striking aspect of this extreme AT-rich genome is the abundance of trinucleotide repeats (predominantly AAT) coding for asparagine [3]. The wealth of low-complexity regions in *P. falciparum* proteins had been known prior to sequencing of the genome but not the overabundance of simple amino acid repeats [4].

## Amino Acid Repeat Prevalence

The random expansion of large amino acid repeats or low-complexity regions in proteomes are usually disfavored. While the effect of these repeats on the structure of the host protein depends on their amino acid compositions, low-complexity regions have a propensity to form loops or disordered structures [5]. Most sequenced genomes have a low abundance of amino acid repeats. However, there are exceptions, notably the social amoeba *Dictyostelium discoideum* and the deadly human malaria parasite *Plasmodium falciparum* [2], [6].

The proteome of *D. discoideum* is rich in polyglutamine and polyasparagine runs of 20 or more residues, found in more than 2,000 proteins [6]. These repeats are overrepresented in certain protein families such as kinases, transcription factors, RNA helicases, and spliceosome components, leading to the suggestion that the expansion of low-complexity regions may be under positive selection [6]. In the case of *P. falciparum*, the repeats are present in about 30% of the proteome and are primarily composed of asparagine residues [7]. The average size of such low-complexity regions is about 37 residues [8]. The asparagine-rich, low-complexity regions in *P. falciparum* are found in all protein families in all developmental stages; they are underrepresented in heat shock proteins and surface antigens [7]. Interestingly, asparagine repeats are rare in other *Plasmodium* species, even though some are as AT-rich as *P. falciparum*. The exception (see below) is the *P. falciparum*–related chimpanzee malaria species *P. reichenowi*, whose genome has not yet been completed, but has nearly identical asparagine repeats where comparisons have been done.

## Consequences of Asparagine Repeats in Proteins

Proteins with large stretches of asparagine repeats have a propensity to form insoluble aggregates, even more so than those with glutamine repeats [9]. Indeed, a large-scale survey of prionogenic proteins in yeast found several proteins that contain asparagine-rich, low-complexity regions and form intracellular aggregates [10]. Formation of insoluble aggregates is not deleterious in all cases [11], [12]. Such protein aggregates have been shown to be important for mediating the inheritance of several phenotypes in yeast [13], persistence of synaptic facilitation in mammals [14], and antiviral innate immunity [15].

In contrast, unregulated aggregation of proteins is harmful and has been associated with various neurodegenerative diseases, systemic amyloidosis, and type II diabetes [12]. Aggregation can lead to cell death by propagating aberrant interactions and by puncturing the cell membrane [16], [17]. In light of this data, the rampant presence of asparagine repeat sequences in 20–30% of all *P. falciparum* proteins is puzzling.

## Heat Shock and Asparagine Repeat–Containing Proteins in *P. falciparum*

The life cycle of *P. falciparum* forces the organism through drastic changes in its environment. Every time the parasite passes from its mosquito vector to its human host and vice versa it faces changing temperatures, with the insect at room temperature and the human at 37°C. A hallmark feature of malaria is cyclical fevers that can last several hours and can exceed 40°C. Heat shock increases the propensity of proteins to unfold and misfold, leading to the formation of aggregates. Given the abundance of asparagine repeat–containing proteins in *P. falciparum*, if all or most of them aggregated during heat shock, it would certainly lead the parasite's demise. However, that is not the case as this is a very successful parasite that has learned to expertly navigate the changing temperature landscape despite the handicap of its asparagine repeat–rich proteome. Learning how the parasite does this holds great promise since it could inform research on how to prevent aggregation of proteins associated with human diseases.

**Figure 1:**
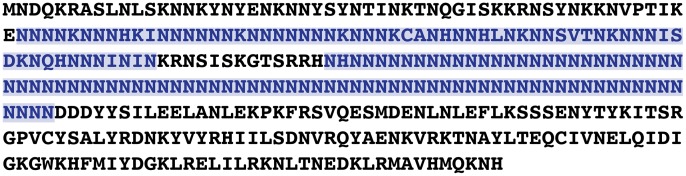
The protein with the largest single continuous stretch of asparagines in the proteome of *P. falciparum*. A protein with distant homology to the regulatory subunit of cell cycle kinase, CDK2, has a stretch of 83 asparagines. Asparagine residues constitute 42% of the primary sequence and it has two asparagine-rich regions (highlighted in blue).

It is possible that the parasite asparagine repeat proteins somehow intrinsically do not aggregate. We tested this hypothesis by expressing a parasite protein that contains an 83-residue asparagine repeat (PF3D7_0923500, Figure 1) in mammalian cells and comparing its aggregation properties to the well-studied asparagine/glutamine-rich prion-forming domain from the yeast translation termination factor, Sup35. Both proteins formed cellular aggregates at 37°C and even more so when incubated at 40°C for a few hours [18]. When both proteins were episomally expressed in *P. falciparum*, no aggregate formation was observed at either temperature, suggesting that parasite chaperones are exceptionally good at preventing aggregation. Indeed, the cytoplasmic *P. falciparum* heat shock protein 110 (*Pf*Hsp110c) was 15 to 30 times better than its yeast or human orthologs at preventing aggregation of asparagine and glutamine repeat proteins in mammalian cells [18]. When the function of *Pf*Hsp110c was knocked down in parasites, they were unable to survive even brief heat shock or prevent aggregation of asparagine repeat–containing proteins [18]. It is likely that *Pf*Hsp110c isn't the only parasite chaperone that is better than its homologs at preventing aggregation. Chaperones always act in concert, so it is possible that other *P. falciparum* heat shock proteins have also evolved to be exceptionally good at preventing protein aggregation. Further research will shed light on this. The data also shows that malarial chaperones may be excellent targets for drug development, as shutting them down will turn the parasite proteome against itself. This work has shown us how the parasite is able to thrive in its fluctuating environment with such a dangerous proteome, but it does not answer why it has such a weird proteome.

## Is There a Functional Role for Asparagine Repeats in *P. falciparum* Proteins?

Why does *P. falciparum* have a proteome abundant in proteins containing asparagine repeats? One obvious answer is that there is a selective advantage or a function that these repeats provide to the parasite. Several hypotheses have been posed. It has been suggested that asparagine repeats act as tRNA sponges [19] or that they have a role in immune evasion and antigenic variation [20], [21] or protein-protein interactions [22]. However, a functional answer seems unlikely since these repeats are present in almost all protein families involved in every metabolic pathway. If there were a specific function for asparagine repeats, they should be enriched in proteins involved in that pathway.

There has been one experimental study that looked at the cellular function of an asparagine repeat in a specific protein. Parasite lines were generated in which a single 28-asparagine repeat in the essential proteasome component, Rpn6, was deleted from the genomic locus [23]. We did not observe any difference between wild-type Rpn6 and the deletion mutant when comparing their expression profiles, protein lifetime, cellular localization, function, and protein-protein interactions. Stressing these parasite lines via heat shock did not reveal any differences either [23]. This suggests that, at least in the case of Rpn6, the asparagine repeat does not have a cellular role.

## Evolution of Asparagine Repeats in the *P. falciparum* Genome

**Figure 2:**
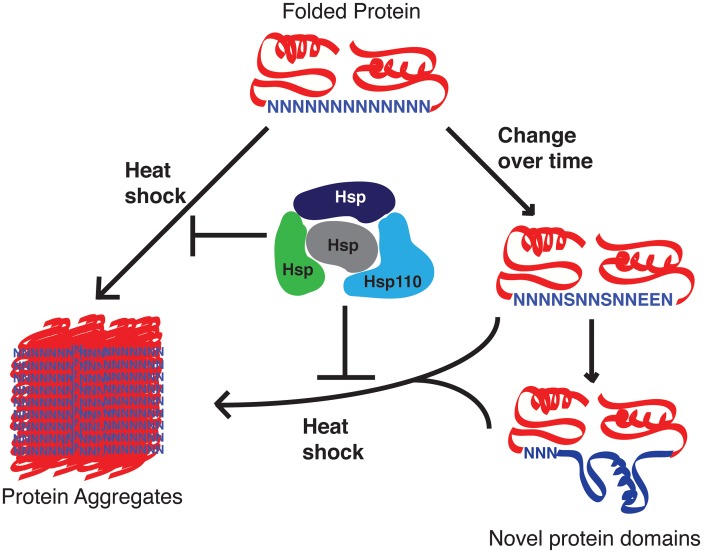
Proposed role of *P. falciparum* Hsp110 and other chaperones as capacitors for evolutionary change. Malaria parasite proteins with asparagine repeat–containing sequences have a greater risk of aggregation. *P. falciparum* Hsp110c, possibly with the help of other chaperones, negates much of this risk, thereby allowing these loop-like regions to mutate. Over time these mutations can give rise to novel protein domains, allowing the parasite to develop new functionalities such as drug resistance and new pathogenic factors.

Bioinformatics analyses suggest that asparagine repeats are primarily being spread in the genome via a DNA-based mechanism, most likely unequal crossover and replication slippage that happens due to the AT-rich nature of the repeats [8], [24]. It is possible then that the abundance of asparagine repeats in the parasite proteome is just an accident of evolution that happened because there is no selective pressure against its propagation. This seems unlikely given the preponderance of evidence showing that asparagine repeat–containing proteins have a greater propensity to aggregate [9] and given the ability of *P. falciparum* to delete unwanted genes and many introns [25]. Others have suggested based on statistical analyses that there is positive selective pressure that specifically promotes the expansion of asparagine repeats within the proteome of *P. falciparum* [26]. Our data suggests that the parasite chaperones are exceptionally good at preventing aggregation and therefore neutralize the negative selective pressure against the expansion of asparagine-rich regions in the proteome of *P. falciparum*. We hypothesize that the *P. falciparum* Hsp110 acts as a capacitor for positive evolutionary change, allowing the propagation of asparagine repeats in the proteome that can then evolve into new protein domains with novel functions (Figure 2) [18], [27]. In the absence of evolutionary and functional evidence, one is left to imagine that repeats like the first one in Figure 1 have started this journey.

It is likely that the last common ancestor of *P. falciparum* and *P. reichenowi* was the origin of these expanded asparagine repeats. Completion of *P. reichenowi* genome sequencing and annotation will shed light on their evolution. Detailed informatics analysis of the few polymorphisms in *P. falciparum* repeats between different strains of this species may also tell us if these features are hotspots for evolutionary change. Understanding the expansion of asparagine repeats will provide vital insights into parasite evolution that could be utilized to tackle this deadly disease.

## References

[1] Cibulskis RE, Aregawi M, Williams R, Otten M, Dye C (2011) Worldwide incidence of malaria in 2009: estimates, time trends, and a critique of methods. PLoS Med 8: e1001142 doi:10.1371/journal.pmed.1001142 22205883 10.1371/journal.pmed.1001142 PMC3243721

[2] Gardner MJ, Hall N, Fung E, White O, Berriman M, et al (2002) Genome sequence of the human malaria parasite Plasmodium falciparum. Nature 419: 498–511. 12368864 10.1038/nature01097 PMC3836256

[3] Aravind L, Iyer LM, Wellems TE, Miller LH (2003) Plasmodium biology: genomic gleanings. Cell 115: 771–785. 14697197 10.1016/s0092-8674(03)01023-7

[4] Kemp DJ, Coppel RL, Anders RF (1987) Repetitive proteins and genes of malaria. Annu Rev Microbiol 41: 181–208. 3318667 10.1146/annurev.mi.41.100187.001145

[5] Wootton JC (1994) Non-globular domains in protein sequences: automated segmentation using complexity measures. Comput Chem 18: 269–285. 7952898 10.1016/0097-8485(94)85023-2

[6] Eichinger L, Pachebat JA, Glöckner G, Rajandream MA, Sucgang R, et al (2005) The genome of the social amoeba Dictyostelium discoideum. Nature 435: 43–57. 15875012 10.1038/nature03481 PMC1352341

[7] Singh GP, Chandra BR, Bhattacharya A, Akhouri RR, Singh SK, et al (2004) Hyper-expansion of asparagines correlates with an abundance of proteins with prion-like domains in Plasmodium falciparum. Mol Biochem Parasitol 137: 307–319. 15383301 10.1016/j.molbiopara.2004.05.016

[8] Zilversmit MM, Volkman SK, DePristo MA, Wirth DF, Awadalla P, et al (2010) Low-complexity regions in Plasmodium falciparum: missing links in the evolution of an extreme genome. Mol Biol Evol 27: 2198–2209. 20427419 10.1093/molbev/msq108 PMC2922621

[9] Halfmann R, Alberti S, Krishnan R, Lyle N, O'Donnell CW, et al (2011) Opposing effects of glutamine and asparagine govern prion formation by intrinsically disordered proteins. Mol Cell 43: 72–84. 21726811 10.1016/j.molcel.2011.05.013 PMC3132398

[10] Alberti S, Halfmann R, King O, Kapila A, Lindquist S (2009) A systematic survey identifies prions and illuminates sequence features of prionogenic proteins. Cell 137: 146–158. 19345193 10.1016/j.cell.2009.02.044 PMC2683788

[11] Fowler DM, Koulov AV, Balch WE, Kelly JW (2007) Functional amyloid – from bacteria to humans. Trends Biochem Sci 32: 217–224. 17412596 10.1016/j.tibs.2007.03.003

[12] Chiti F, Dobson CM (2006) Protein misfolding, functional amyloid, and human disease. Annu Rev Biochem 75: 333–366. 16756495 10.1146/annurev.biochem.75.101304.123901

[13] Patino MM, Liu JJ, Glover JR, Lindquist S (1996) Support for the prion hypothesis for inheritance of a phenotypic trait in yeast. Science 273: 622–626. 8662547 10.1126/science.273.5275.622

[14] Si K, Choi Y-B, White-Grindley E, Majumdar A, Kandel ER (2010) Aplysia CPEB can form prion-like multimers in sensory neurons that contribute to long-term facilitation. Cell 140: 421–435. 20144764 10.1016/j.cell.2010.01.008

[15] Hou F, Sun L, Zheng H, Skaug B, Jiang Q-X, et al (2011) MAVS forms functional prion-like aggregates to activate and propagate antiviral innate immune response. Cell 146: 448–461. 21782231 10.1016/j.cell.2011.06.041 PMC3179916

[16] Olzscha H, Schermann SM, Woerner AC, Pinkert S, Hecht MH, et al (2011) Amyloid-like aggregates sequester numerous metastable proteins with essential cellular functions. Cell 144: 67–78. 21215370 10.1016/j.cell.2010.11.050

[17] Seuring C, Greenwald J, Wasmer C, Wepf R, Saupe SJ, et al (2012) The mechanism of toxicity in HET-S/HET-s prion incompatibility. PLoS Biol 10: e1001451 doi:10.1371/journal.pbio.1001451 23300377 10.1371/journal.pbio.1001451 PMC3531502

[18] Muralidharan V, Oksman A, Pal P, Lindquist S, Goldberg DE (2012) Plasmodium falciparum heat shock protein 110 stabilizes the asparagine repeat-rich parasite proteome during malarial fevers. Nat Commun 3: 1310. 23250440 10.1038/ncomms2306 PMC3639100

[19] Frugier M, Bour T, Ayach M, Santos MAS, Rudinger-Thirion J, et al (2010) Low complexity regions behave as tRNA sponges to help co-translational folding of plasmodial proteins. FEBS Lett 584: 448–454. 19900443 10.1016/j.febslet.2009.11.004

[20] Verra F, Hughes AL (1999) Biased amino acid composition in repeat regions of Plasmodium antigens. Mol Biol Evol 16: 627–633. 10335656 10.1093/oxfordjournals.molbev.a026145

[21] Hughes AL (2004) The evolution of amino acid repeat arrays in Plasmodium and other organisms. J Mol Evol 59: 528–535. 15638464 10.1007/s00239-004-2645-4

[22] Karlin S, Brocchieri L, Bergman A, Mrazek J, Gentles AJ (2002) Amino acid runs in eukaryotic proteomes and disease associations. Proc Natl Acad Sci U S A 99: 333–338. 11782551 10.1073/pnas.012608599 PMC117561

[23] Muralidharan V, Oksman A, Iwamoto M, Wandless TJ, Goldberg DE (2011) Asparagine repeat function in a Plasmodium falciparum protein assessed via a regulatable fluorescent affinity tag. Proc Natl Acad Sci U S A 108: 4411–4416 doi:10.1073/pnas.1018449108 21368162 10.1073/pnas.1018449108 PMC3060247

[24] Xue HY, Forsdyke DR (2003) Low-complexity segments in Plasmodium falciparum proteins are primarily nucleic acid level adaptations. Mol Biochem Parasitol 128: 21–32. 12706793 10.1016/s0166-6851(03)00039-2

[25] Anderson TJC, Patel J, Ferdig MT (2009) Gene copy number and malaria biology. Trends Parasitol 25: 336–343. 19559648 10.1016/j.pt.2009.04.005 PMC2839409

[26] Dalby AR (2009) A comparative proteomic analysis of the simple amino acid repeat distributions in Plasmodia reveals lineage specific amino acid selection. PLoS ONE 4: e6231 doi:10.1371/journal.pone.0006231 19597555 10.1371/journal.pone.0006231 PMC2705789

[27] Rutherford SL, Lindquist S (1998) Hsp90 as a capacitor for morphological evolution. Nature 396: 336–342. 9845070 10.1038/24550

